# Fibula Pro-tibia or Tibial Pro-fibula Dilemma: Order Defines Meaning

**DOI:** 10.5704/MOJ.2411.014

**Published:** 2024-11

**Authors:** RY Kow, CL Low, N Mohd-Yusof

**Affiliations:** 1 Department of Orthopaedics, Traumatology and Rehabilitation, International Islamic University Malaysia, Kuantan, Malaysia; 2 Department of Radiology, International Islamic University Malaysia, Kuantan, Malaysia

Dear editor,

We read the excellent article by Jain *et al*^1^ with avid interest. The authors successfully managed complex distal tibia and fibula fractures with the rarely-used fibula pro-tibia fixation technique, achieving good or excellent outcomes in 29 out of 30 patients. In fact, this technique was first described by Campanacci and Zanoli in 1966 to treat non-union of the tibia by creating a fibular synostosis^2^. Since then, a few authors have used the technique to treat ankle fractures in high-risk patients, such as those with osteoporotic bone or diabetes mellitus^2,3^.

**Fig. 1:**
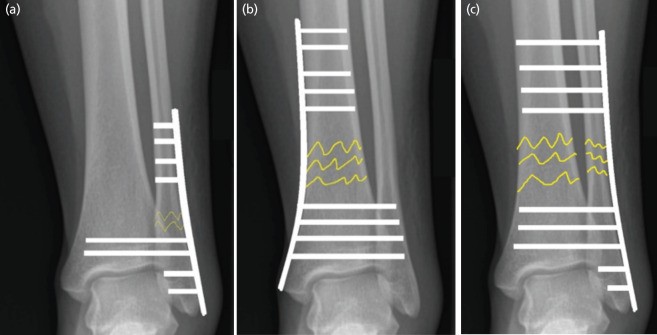
(a) In this patient with fibula fracture, the screws are transfixed onto the metaphysis of the tibia to enhance construct stability. Hence, this construct should be called tibia pro-fibula, as the tibia supports the fibula fixation. (b) In patients with tibial fracture, the screws are transfixed onto the distal fibula to obtain better mechanical support. In this scenario, the construct can be called fibula pro-tibia. (c) In this case, the construct can be termed as fibula pro-tibia, as the fibula is in support of the tibia.

This intriguing title raises the question: is it termed fibula pro-tibia or tibial pro-fibula, as both are reported in the literature?^1-4^. To answer this question, one must delve into basic English grammar. The term “A pro-B” means “A in favour of B” or “A supporting B”. When this principle is applied, fibula pro-tibia means the fibula is supporting the tibia, and vice versa for tibia pro-fibula. For example, in Fig. 1a, where there is a fibula fracture, the screws are transfixed onto the metaphysis of the tibia to enhance construct stability. Hence, this construct should be called tibia pro-fibula, as the tibia supports the fibula fixation. The clinical cases are described in detail by Panchbhavi *et al*^3^. Confusion exists even in the literature, where Okoro *et al* incorrectly described their construct as fibula pro-tibia, instead of tibia pro-fibula^4^. In other words, it should not be based on the plate’s location, but rather on the function of the supporting bone.

In contrast, in a tibial fracture model depicted in Fig. 1b, the screws are transfixed onto the distal fibula to obtain better mechanical support. In this scenario, the construct can be called fibula pro-tibia, with the clinical cases published by Said *et al*^2^. In the article by Jain e*t al*, they transfixed the screws onto the tibia from the fibula plate to address both distal tibia and fibula fractures (Fig. 1c)^1^. In this case, the title is correctly termed as fibula pro-tibia, as the fibula is in support of the tibia.

Based on the suggestion by Reudi and Allgower^5^, the management of distal tibia and fibula fractures should be: (1) reduction and fixation of fibula; (2) reduction of the tibia articular surface; (3) grafting of the metaphyseal defect; and (4) medial fixation of tibia. Using this fibula pro-tibia fixation construct, steps 2 to 4 can be skipped all together. Additionally, by minimising the surgical incision, the soft tissue violation and vascular compromise can be minimised, which may help achieve bony union.

Nevertheless, any surgeon should carefully weigh the surgical options before pursuing this method for treating complex distal tibia and fibula fractures. While the authors successfully immobilise the tibia fracture during fibula fixation with Kirschner wires, it is easier said than done, especially for junior surgeons. The difficulty is compounded by intraarticular tibial fractures. Multiple attempts at manipulation and reduction may prolong the surgery and potentially damage the surrounding soft tissues and vasculature, making this method counterintuitive. On top of that, using a single plate to address both distal tibia and fibula fractures is biomechanically inferior compared to fixation using double or triple plates. Hence, this method has only been used in high-risk patients. Furthermore, prolonged post-operative immobilisation and extended duration of non-weight bearing owing to inferior biomechanics strength may lead to ankle stiffness, especially in young and active individuals. Similarly, with the transfix screws in place, ankle stiffness may occur, though this phenomenon was not observed in the series reported by Jain *et al*^1^.

While we applaud the excellent work by Jain *et al*^1^, whether this “internal external fixator” is superior to conventional plating or traditional external fixation requires further research.
