# Supplementary material for: Fibula Pro-tibia or Tibial Pro-fibula Dilemma: Order Defines Meaning
Source: Malays Orthop J. 2024 Nov;18(3):88–9. doi: 10.5704/MOJ.2411.014 (PMC11647536; doi:10.5704/MOJ.2411.014)
Supplement: Related texts

# Authors’ Reply to the Letters to the Editor

**Published:** 2024-11

**Authors:** Saurabh Jain, Anand Ajmera

**Affiliations:** Department of Orthopaedics, Mahatma Gandhi Memorial Government Medical College, Indore, India

Thanks for reading our article and appreciating it. Our study demonstrated good to excellent outcomes of fibula pro-tibia plating in complex distal tibia fractures. The study was conducted on 30 patients between patients of age 18 to 60 years in cases of distal tibia fibula fractures with tibial fracture line extending within 5cm from the tibial plafond with or without the involvement of articular surface presenting within a week of injury. It was further stated that patients with open tibial fractures i.e. wound over medial or anterior part of leg, were included whereas patients having open fibular fractures of Gustilo Anderson grade 3B and above not amenable to fibular plate fixation were excluded from the study. Hence to summarise, inclusion criteria in our study particularly included patients of distal tibia and fibula fractures with open wound on medial/anterior (tibial) side which were relatively unfit for medial tibia plating^1^.

These patients of distal tibia fibula fractures having wound on medial or anterior side were managed with fibula pro-tibia plating. In this technique, the first step involved was same as given by Ruedi and Allgower i.e. fibular reduction and plate fixation^2^. It is same standard step as done in any other treatment protocol of distal tibial fractures, not requiring anything new or deviation from basic principles. But this does not mean that other steps as suggested by Ruedi and Allgower which are equally important, were skipped by the fibula pro-tibia plating technique. Technique still follows the principles and other steps of the management, as it involves the reduction of distal tibial articular surface via indirect means and provisional fixation with k wires. Since these patients had medially or anteriorly open wound on tibia, bone grafting was not done for these cases. Further the tibia fixation was achieved indirectly via the fibular cortical strut, locking fibular plate and fibula pro-tibia screws with each screw having four cortical hold (both fibula and tibia) providing very high strength to the construct which can very well hold the tibial fracture fragments in reduced and aligned position. Thus, fibula pro-tibia plating has advantage of stabilising the both lateral and medial pillar by a single plate and leaving the medial tibial soft tissue sleeve and periosteum intact and preventing any surgical injury to the tenuous tibial blood supply, skin and soft tissue surroundings on the tibia medially, which will help in early union avoiding wound related complications associated with medial plating. Further application of just one plate laterally on fibula in comparison to two plates for both fibula and tibia, will also decrease the surgical time, blood loss, radiation exposure, infection rate and cost^1^.

The longer screws fixing fibula and tibia are almost the same as syndesmotic screws. With fibula pro-tibia plating, theoretically there is risk of limitation of ankle range of motion and screw breakage like syndesmotic screws failure occurs if not removed. But since all the screws in the technique are fibula pro-tibia screws, the stresses on the individual screws are distributed unlike the syndesmotic single screw and thus it prevents breakage. Further placing these screws with ankle in maximal dorsiflexion prevents the narrowing of ankle mortise and hence preserves ankle mobility. We believe that ankle stiffness is not due to the technique of fibula pro-tibia plating, but primarily due to complication arising due to initial trauma and involvement of the articular margin per se.

We agree with the concern of the authors that the reduction of the distal tibia fracture is difficult, and this technique has a learning curve. Maybe we have gained experience and expertise after some initial cases which might have helped us in reducing the fracture and treating these patients with ease. We suggest surgeons to gain expertise and experience before attempting to treat these cases with the technique of fibula pro-tibia plating.

We also accept the fact that the single plate fixing both the tibia and fibula may be biomechanical weaker construct than dual plate construct of fixing both fibula and tibia by lateral fibular and medial tibial plates respectively. But we advocate the technique of fibula pro-tibia plating specially in cases of distal tibia and fibula fractures with open wound on tibial side, who are relatively contraindicated for medial tibial plating due to high risk for infection if plated on tibia. We do not propagate the technique as superior or alternative to medial tibia plating, but we propose it to be a treatment option for cases, where the medial plating cannot be done. Further comparative studies or higher level of evidence studies are needed to evaluate this further.

Regarding the authors concern of using the term “fibula – pro-tibia”, we are of the opinion that ‘fibula pro-tibia’ is a better term to describe the technique as mentioned by Said *et al*^3^ and DeOrio *et al*^4^ compared to the term ‘tibia pro-fibula’ as stated by Panchbhavi *et al*^5^. As the authors have delved into the literature and stated that term “A pro-B” means “A in favour of B” or “A supporting B”. But the authors have misinterpreted the concept by mentioning it as tibia supporting fibula, whereas actually it is the fibula that is supporting the tibia. In the technique used in our study, there is no fixation done on tibia side and the tibia is being supported by the fibula via fibula plate fixation and all screws of the fibula plate passing and holding the tibia fracture also, thus supporting tibia. Hence fibula pro-tibia plating seems to be a more appropriate term rather than tibia pro-fibula.

Lastly, we agree to the fact that further research is required to validate the results and generate a higher level of evidence; hence we have added the term, a pilot study in the title itself. We hope that further multicentre studies, with larger cohort and longer follow-ups can answer the controversies aroused.

## References

[1] Jain S, Khare H, Verma K, Kumar U, Ajmera A (2024). How Effective is Fibula Pro Tibia Plating in Treatment of Distal Tibial Fractures: A Pilot Study.. Malays Orthop J..

[2] Rüedi TP, Allgöwer M (1979). The operative treatment of intra-articular fractures of the lower end of the tibia.. Clin Orthop Relat Res..

[3] Said GZ, El-Sharkawi MM, Said HG, Refai OA (2011). Fibula-pro-tibia in plating tibial non-unions.. Int Orthop..

[4] DeOrio JK, Ware AW (2003). Salvage technique for treatment of periplafond tibial fractures: the modified fibula-pro-tibia procedure.. Foot Ankle Int..

[5] Panchbhavi VK, Mody MG, Mason WT (2005). Combination of hook plate and tibial pro-fibular screw fixation of osteoporotic fractures: a clinical evaluation of operative strategy.. Foot Ankle Int..
